# The impact of health education videos on general public’s mental health and behavior during COVID-19

**DOI:** 10.1186/s41256-021-00211-5

**Published:** 2021-09-30

**Authors:** Qian Yang, Zhihua Wu, Ying Xie, Xiaohua Xiao, Jinnan Wu, Tian Sang, Kejun Zhang, Haidong Song, Xifeng Wu, Xin Xu

**Affiliations:** 1 Center for Health Policy Studies, School of Public Health and Department of Endocrinology of The Children’s Hospital, National Clinical Research Center for Child Health , Zhejiang University School of Medicine, Hangzhou, 310058 China; 2 Department of Public Health, Zhejiang University School of Medicine, Hangzhou, China; 3 Department of Graduate School of Education and Psychology, Pepperdine University, California, USA; 4 College of Computer Science and Technology, Zhejiang University, Hangzhou, China; 5 Hangzhou Seventh People’s Hospital, Hangzhou, China; 6 Memory, Ageing and Cognition Centre, Department of Pharmacology, National University of Singapore, Singapore, Singapore

**Keywords:** COVID-19, Health education, Public health, Health-related behavior

## Abstract

**Background:**

COVID-19 has seriously affected people's mental health and changed their behaviors. Previous studies for mental state and behavior promotion only targeted limited people or were not suitable for daily activity restrictions. Therefore, we decided to explore the effect of health education videos on people’s mental state and health-related behaviors.

**Methods:**

Based on WeChat, QQ, and other social media, we conducted an online survey by snowball sampling. Spearman’s non-parametric method was used to analyze the correlation related to mental health problems and health-related behaviors. Besides, we used binary logistic regression analyses to examine mental health problems and health-related behaviors' predictors. We performed SPSS macro PROCESS (model 4 and model 6) to analyze mediation relationships between exposure to health education videos and depression/anxiety/health-related behaviors. These models were regarded as exploratory.

**Results:**

Binary logistic regression analyses indicated that people who watched the health education videos were more likely to wear masks (OR 1.15, *p* < 0.001), disinfect (OR 1.26, *p* < 0.001), and take temperature (OR 1.37, *p* < 0.001). With higher level of posttraumatic growth (PTG) or perceived social support (PSS), people had lower percentage of depression (For PSS, OR 0.98, *p* < 0.001; For PTG, OR 0.98, *p* < 0.01) and anxiety (For PSS, OR 0.98, *p* < 0.001; For PTG, OR 0.98, *p* = 0.01) and better health behaviors. The serial multiple-mediation model supported the positive indirect effects of exposure to health education videos on the depression and three health-related behaviors through PSS and PTG (Depression: B[SE] =  − 0.0046 [0.0021], 95% CI − 0.0098, − 0.0012; Mask-wearing: B[SE] = 0.0051 [0.0023], 95% CI 0.0015, 0.0010; Disinfection: B[SE] = 0.0059 [0.0024], 95% CI 0.0024, 0.0012; Temperature-taking: B[SE] = 0.0067 [0.0026], 95% CI 0.0023, 0.0013).

**Conclusion:**

Exposure to health education videos can improve people's self-perceived social support and inner growth and help them cope with the adverse impact of public health emergencies with better mental health and health-related behaviors.

**Supplementary Information:**

The online version contains supplementary material available at 10.1186/s41256-021-00211-5.

## Background

At the end of 2019, the Coronavirus Disease 2019 (COVID-19) pandemic broke out and spread globally. The World Health Organization (WHO) announced COVID-19 as a global pandemic at the media briefing on March 11th, 2020. This pandemic not only seriously threatened the lives of people around the world, but also had a profound impact on people’s mental health and health behavior [1–3]. An investigation at the beginning of the COVID-19 pandemic in China showed that more than half of the respondents believed they suffered from moderate to severe psychological problems from the pandemic. Approximately one-third of the respondents reported they were experiencing moderate to severe anxiety [4]. A Meta-analysis of the psychological states among different populations in the COVID-19 pandemic indicated that 72.9% of the participants had suffered mental health problems, including anxiety and depression, which were particularly severe [5, 6]. In addition, the novel coronavirus 2019 is strong in transmission and highly occult; people are generally susceptible to the disease. In the initial stage of the COVID-19 outbreak, health-related behaviors like home quarantine, mask-wearing, disinfection, and temperature-taking are vital for prevention. For instance, face masks can reduce exposure to airborne viruses [7], while reducing travel and staying at home are basic means accepted by health authorities [8]. Thus, both mental health and health-related behavior promotion should be taken seriously during the pandemic.

Psychological and behavioral intervention is an important part of the response to public health emergencies. To contain the adverse effects that the COVID-19 pandemic had on individuals and society, we need to strengthen psychological counseling and behavioral intervention for the whole population. Chinese Center for Disease Control and Prevention issued “Guiding Principles for Emergency Psychological Crisis Intervention in Response to Novel Coronavirus Pneumonia Epidemic” on January 26th, 2020, which clarifies basic principles and intervention points for psychological crisis intervention and psychological counseling during the epidemic. Besides, the Chinese government has launched a series of prevention and control measures to contain the spread of the virus and released the Public Prevention Guide for Novel Coronavirus Infected Pneumonia, which advises the public to adopt a series of health behaviors to prevent the disease. Under such circumstances, the School of Public Health of Zhejiang University produced eight health education videos and one tweet for the public in China. These products have been published on various e-platforms such as People’s Daily Online, Weibo, QQ, and other mainstream media since January 27th, 2020, attracting extensive attention from the public.

During public health emergencies, the scientific popularization of public health plays a vital role in health education, and psychological and behavioral intervention [9]. However, some classic interventions, like meetings or offline courses, could not be implemented during the pandemic due to home quarantine. Video-based health education became a critical way to do so. However, the research on video-based health popularization mainly focuses on improving participants' knowledge or attitude. Few studies investigate the influence of mental health and behaviors [10, 11]. A systematic review showed that, while video-based educational interventions have been used to promote specific preventive health behaviors, significant improvements in behavioral outcomes were not reported uniformly across all studies [12]. Therefore, it is important to evaluate video-based health education that promote health behaviors during the COVID-19 pandemic. The health education videos we provided encouraged the public to keep a calm mind, not to be afraid or panic, taught ways to relieve negative emotions, taught people how to behave appropriately, and emphasized the benefits of adopting health-related behaviors to individuals and society.

As a major life trauma, COVID-19 may negatively affect individuals, such as posttraumatic stress symptoms. Many studies have examined the negative psychological effects of COVID-19. Still, we need to focus on the positive effects of negative events. Studies have shown that, during a crisis, one could stay hopeful by paying attention to optimistic information and mitigating individuals' pessimistic psychological problems [13], with the positive function adaptation for life challenges [14]. Posttraumatic growth (PTG) is a critical factor in promoting health behaviors [15], which means positive change experienced due to the struggle with a major life crisis or a traumatic event [16]. However, few studies have focused on how adverse situations can produce positive outcomes, let alone health education approaches that promote PTG [17]. Our health education videos spread scientific information, delive red positive messages to people, such as that we are bound to win the war against COVID-19. Besides, our health education videos helped sc reen out false information, such as drinking hotwater, taking asauna, or turning on heating can kill coronavirus.

Additionally, we encouraged people to take the initiative to contact the community and provided psychological assistance hotline and CDC consultation phone numbers. It may, to some extent, help the public increase perceived social support (PSS). PSS means an individual’s subjective perceived support ties with other individuals, groups, and the larger community. PSS protects lonely people in daily life and during unexpected disasters [18]. A study on the psychosocial health of left-behind children found that psychosocial health was positively correlated with social support, and psychosocial health may be affected by subjective support [19]. Social factors can influence behaviors, and social support is one of the factors that influence participants' health-related behaviors [20–22].

Previous studies have shown that PTG is significantly positively correlated with PSS [23]. A study of 4,170 high school students in China who experienced the Wenchuan earthquake found that the more social support they received from family, teachers, friends, and other sources, the higher their PTG scores [24]. Based on the discussion mentioned above, we proposed the following hypotheses: Exposure to health education videos is negatively related to mental health problems (Anxiety and Depression), and positively related to health-related behaviors (Home quarantine, Mask-wearing, Disinfection, and Temperature-taking). Beside, PTG and PSS were mediators of the relationship between exposure to health education videos and mental health problems /health-related behaviors, and they have serial multiple mediation effects.

This study aims to provide new public health education strategies and recommendations for policy-making, which may contribute to the prevention of mental disorders and health-related behaviors. We explored the effects of exposure to health education videos in promoting mental health (including anxiety, depression and PTG) as well as the health-related behaviors (including mask-wearing, temperature-taking, disinfection and home quarantine) and the possible processes.

## Methods

### Participants

A total of 2710 participants from 30 provinces or regions across China were investigated. The inclusion criteria were 18 years and over in age, living in China, and sufficient command over Chinese. The exclusion criteria were age over 80 years old and failing in the attention test. In China, most of the elderly over 80 years old do not know how to use smartphones. Since this study adopted electronic questionnaires, they were excluded.

## Research design

This study included eight health education videos and one tweet produced by the School of Public Health of Zhejiang University targeting the COVID-19 pandemic. Most of these products are short videos, mainly in a vivid animation, and popularize the widespread scientific knowledge of COVID-19 prevention to the public. These health education videos/tweets: (1) introduce Novel Coronavirus 2019 and its features, and measures conducive to personal protection (home isolation, wearing masks, seven-step hand-washing method, food safety, etc.). (2) provide ways to alleviate anxiety during COVID-19. (3) uncover rumors about Novel Coronavirus 2019 and offer telephone numbers of CDC and psychological counseling, and the like. All products are available for free on the Internet (See Additional file 1: Table S1 for main contents and network links for each product).

### Questionnaire development

The questionnaire includes the following components: (a) Basic demographics (such as gender, age, and current living area). Current living area is a categorical variable, which is divided into 5 categories according to the number of confirmed cases in the region, respectively high-risk areas (≥ 2000 confirmed cases), high risk (1000–1999 confirmed cases), moderate-high risk (500–599 confirmed cases), moderate risk (100–499 confirmed cases) and Mild-moderate risk (< 100 confirmed cases); (b) The total number of the health education videos/tweets from Zhejiang University they had watched; (c) The health-related behavior (mask-wearing, temperature-taking, disinfection, and home quarantine); (d) Primary source of information. We set up 11 kinds of information sources and divided them into two categories based on whether they were published by official agencies or not. If the participant's primary source of information is official media accounts on social media (e.g. CCTV) / mainstream media websites/client-sides (e.g. China Central Television) / official face-to-face communication (e.g., community broadcasting) / TV programs (national or local TV stations) / newspapers in print (national or local newspaper) / traditional communication tools (e.g. SMS alerts from CDC), we believe that the primary source of information is reliable. Suppose it is other We-Media public accounts on social media / personal accounts on social media (e.g. Weibo) / network communication tools (e.g. WeChat) / short video platforms (e.g. Tik Tok) / face-to-face communication in life. In that case, the primary information source is considered unreliable; (e) Psychological scales to evaluate the participants’ psychological state. The following scales were used:

1. Patient Health Questionnaire-9, PHQ-9 [25]: assesses subject’s depression level in the past two weeks. There are nine items in total (e.g., “Little interest or pleasure in doing things”). Each item is 0–3 points (0-not at all or very little, 1-sometimes, 2-considerable amount of time, 3-most or all the time), the total score is 0–27. Scoring standard: 0–4 for no depression, 5–27 for depression.
2. General Anxiety Disorder-7, GAD-7 [26]: assesses subject’s anxiety level in the past two weeks. There are seven items in total (e.g., “Feeling nervous, anxious or on edge”). Each item is 0–3 points (0-not at all or very little, 1-sometimes, 2-a considerable amount of time, 3-most or all the time). Scoring standard: 0–4 for no anxiety, 5–21 for anxiety.
3. Posttraumatic Growth Inventory, PTGI [16]: measures the positive psychological changes that result from fighting against traumatic and negative life events and situations. There are ten items in total (e.g., “I changed my priorities about what is important in life”). It uses a 7-level scoring method, with one deleted item due to cultural differences, ranging from “not at all to” “a very great degree” (1–7 points respectively), with a total score of 9–63 points. Higher scores indicate a higher level of PTG.
4. Perceived Social Support Scale, PSSS [27]: emphasizes an individual’s self-understanding and self-perceived social support. There are 12 items in total (e.g., “There is a special person who is around when I am in need”). It uses a 7-level scoring method ranging from “strongly disagree” to “strongly agree” (1–7 points respectively), with a total score of 12–84 points. Higher scores indicate a higher PSS.

All of the scales indicated high reliability—PHQ-9 (α = 0.927), GAD-7 (α = 0.944), PTGI (α = 0.939), PSSS (α = 0.962).

### Data collection

Data were extracted from an online survey from February 13 to February 15, 2020. Before distributing the questionnaires, we used the snowball sampling technique to find the WeChat groups and QQ groups of Chinese residents from all provinces and regions, and sent our health education videos and tweets to them. Participants decided whether or not to view these products, since it was not mandatory. An informed consent form accompanied the e-questionnaire, and each participant was required to submit it before completing the e-questionnaire. The most familiar social media platforms in China, WeChat and QQ, were used for distribution. To prevent the participants not filling in the questionnaire carefully, we set up an attention test: this question is to check whether you are paying attention to the questions. Please choose the second option, "very little". If participants did not select the option "very little", the questionnaire is considered invalid. At last, a total of 2685 participants were included in the analysis.

### Statistical analysis

All statistical analyses were performed with SPSS 24.0. Descriptive analyses were conducted for all demographic data and questionnaire scores. For the continuous variables, medians and interquartile ranges were used, whereas categorical data were presented as numbers and percentages. Then, for correlation analysis, Spearman’s non-parametric method was performed with mental health problems and health-related behaviors as binary variables. Moreover, we conducted binary logistic regression analyses to examine mental health problems and health-related behavior predictors. Finally, the SPSS macro PROCESS was used to analyze mediation relationships between exposure to health education videos and depression/anxiety/health-related behaviors. Age, sex, current living area, survey date, and primary source of information were entered as co-variants in all mediation analyses. We tested more complex mediation models involving two mediators employing SPSS macro PROCESS. These models were regarded as exploratory. The statistical significance level for all the tests was set at a *p *value of below 0.05.

## Results

A total of 2710 participants completed the survey, among whom five were not living in China, 18 were over 80 years old, and two had repeated answers (removed from the dataset), leaving a total of 2685 in the current analysis. All 2685 participants completed all questionnaires in the survey. Descriptive statistics for all variables are presented in Table 1.

**Table 1:** Descriptive statistics for all variables (N = 2685)

Variables	Values, median (IQR) OR n (%)
Age (years), median (IQR)	27 (22.33)
Gender, n (%)	
Male	1613 (60.1)
Female	1072 (39.9)
Current living area, n (%)	
Extremely high risk (≥ 2000 confirmed cases)	98 (3.6)
High risk (1000–1999 confirmed cases)	594 (22.1)
Moderate-high risk (500–599 confirmed cases)	680 (25.3)
Moderate risk (100–499 confirmed cases)	1164 (43.4)
Mild-moderate risk (< 100 confirmed cases)	149 (5.5)
Primary source of information, n (%)	
Reliable	2044 (76.1)
Unreliable	641 (23.9)
Exposure to health education videos, median (IQR)	3 (2.4)
Perceived Social Support (PSSS total score), median (IQR)	64 (55.73)
Posttraumatic growth (PTGI total score), median (IQR)	41 (35.46)
Depression, n (%)	
No (PHQ total score ≤ 4)	861 (32.1)
Yes (PHQ total score > 4)	1824 (67.9)
Anxiety, n (%)	
No (GAD total score ≤ 4)	1225 (45.6)
Yes (GAD total score > 4)	1460 (54.4)
Home quarantine, n (%)	
> 1time	659 (24.5)
0–1 times	2026 (75.5)
Mask-wearing, n (%)	
No	544 (20.3)
Yes	2141 (79.7)
Disinfection, n (%)	
No	641 (23.9)
Yes	2044 (76.1)
Temperature-taking, n (%)	
No	537 (20.0)
Yes	2044 (80.0)

### Correlations between mental health problems, health-related behaviors, and exposure to health education videos

Table 2 displays non-parametric Spearman correlations between exposure to health education videos, PSSS, PTGI, mental health problems, and health-related behaviors. It indicates that exposure to health education videos correlated positively with PSSS, PTGI, and health-related behaviors. PSSS and PTGI scores correlated positively with all health-related behaviors (Home quarantine, mask-wearing, disinfection, and temperature-taking), while correlated negatively with depression and anxiety. Anxiety correlated positively with mask-wearing and temperature-taking.

**Table 2:** Means, Standard Deviations, correlations among exposure to health education videos, PSSS, PTGI, mental health problems and health-related behaviors (N = 2685)

	M	SD	1	2	3	4	5	6	7	8	9
1. Exposure to health education videos	3.06	2.11	—								
2. Perceived Social Support Scale	62.79	14.48	0.089***	—							
3. Posttraumatic Growth Inventory	40.09	9.10	0.199***	0.681***	—						
4. Depression	0.68	0.54	− 0.016	− 0.256***	− 0.228***	—					
5. Anxiety	0.47	0.50	0.036	− 0.248***	− 0.193***	0.648***	—				
6. Home quarantine	0.75	0.13	0.017	0.069***	0.029	0.003	0.001	—			
7. Mask-wearing	0.80	0.10	0.184***	0.085***	0.159***	0.037	0.065**	− 0.010	—		
8. Disinfection	0.76	0.13	0.255***	0.157***	0.227***	− 0.010	0.038	0.042*	0.398***	—	
9. Temperature-taking	0.80	0.10	0.297***	0.129***	0.218***	0.934	0.062**	0.018	0.404***	0.513***	—

Categorical variables were dichotomized and coded as 0 and 1 to aid in interpretation. Depression (0 = no depression; 1 = depression); Anxiety (0 = no anxiety; 1 = anxiety); Home quarantine (0 = go out more than 1 time in the last 3 days, 1 = go out less than 1 time in the last 3 days.); Mask-wearing (0 = no wearing mask, 1 = wearing mask.); Disinfection (0 = no disinfection, 1 = disinfection.) Temperature-taking (0 = no taking temperature, 1 = taking temperature.)

*p < 0.05; **p < 0.01; ***p < 0.001

### Binary logistic regression

To assess the factors associated with mental health problems and health-related behaviors during COVID-19, we ran binary logistic regression models, including all variables. The logistic regression analysis results are summarized in Table 3.

**Table 3:** Predictors of mental health problems and health-related behaviors during COVID-19 (N = 2685)

variables	Mental health problems		Health-related behaviors			
	Y = Depression	Y = Anxiety	Y = Home quarantine	Y = Mask-wearing	Y = Disinfection	Y = Temperature-taking
	Odds ratio (95% CI)	Odds ratio (95% CI)	Odds ratio (95% CI)	Odds ratio (95% CI)	Odds ratio (95% CI)	Odds ratio (95% CI)
Exposure to health education videos	0.977 (0.938, 1.019)	1.008 (0.973, 1.049)	1.019 (0.978, 1.066)	1.149*** (1.089, 1.213)	1.256*** (1.189, 1.327)	1.365*** (1.280, 1.456)
Perceived Social Support Scale	0.977*** (0.969.0.986)	0.978*** (0.970, 0.986)	1.012** (1.004, 1.021)	1.009 (1.00, 1.018)	1.015** (1.006, 1.024)	1.013** (1.003, 1.023)
Posttraumatic Growth Inventory	0.977** (0.963, 0.990)	0.984* (0.972, 0.996)	0.993 (0.979, 1.006)	1.018* (1.004, 1.033)	1.024** (1.009, 1.0390	1.027** (1.011, 1.043)
Age	0.966*** (0.957, 0.976)	0.979*** (0.970, 0.989)	0.993 (0.983, 1.003)	1.032*** (1.019, 1.045)	1.031*** (1.019, 1.044)	1.013* (1.000, 1.025)
Gender (ref: Female)	1.097 (0.921, 1.308)	1.075 (0.911, 1.268)	0.624*** (0.515, 0.756)	1.581*** (1.286, 1.944)	0.953 (0.776, 1.169)	0.986 (0.788, 1.233)
Current living area (ref. extremely high risk)						
High risk	0.798 (0.497, 1.282)	0.855 (0.549, 1.333)	0.685 (0.390, 1.204)	1.505 (0.920, 2.462)	1.047 (0.627, 1.750)	0.927 (0.527, 1.630)
Moderate-high risk	0.670 (0.420, 1.069)	0.690 (0.445, 1.070)	0.658 (0.377, 1.148)	1.774* (1.088, 2.891)	1.151 (0.693, 1.912)	1.015 (0.581, 1.771)
Moderate risk	0.999 (0.633, 1.578)	1.036 (0.676, 1.587)	0.773 (0.447, 1.336)	2.417*** (1.498, 3.900)	1.793* (1.087, 2.956)	1.543 (0.890, 2.676)
Mild-moderate risk	1.109 (0.618, 1.989)	1.039 (0.608, 1.776)	0.637 (0.335, 1.211)	2.106* (1.118, 3.969)	1.540 (0.817, 2.904)	1.269 (0.632, 2.547)
Primary source of information (ref. Unreliable)	0.786* (0.641, 0.966)	0.759** (0.629, 0.916)	1.209 (0.986, 1.483)	0.821 (0.645, 1.046)	1.130 (0.904, 1.413)	0.964 (0.751, 1.237)

*p < 0.05; **p < 0.01, ***p < 0.001

Participants who watched more science videos were more likely to wear masks (OR 1.15, *p* < 0.001), disinfect (OR 1.26, *p* < 0.001), and take temperature (OR 1.37, *p* < 0.001). Compared with participants who perceived low social support, those with high social support were less likely to be depressed (OR 0.98, *p* < 0.001) and anxious (OR 0.98, *p* < 0.001), and more likely to stay at home (OR 1.01, *p* < 0.01), disinfect (OR 1.02, *p* < 0.01) and take temperatures (OR 1.01, *p* < 0.01). Compared with participants with a lower level of PTG, participants with high-level PTG were less likely to be depressed (OR 0.98, *p* < 0.01) and anxious (OR 0.98, *p* = 0.01), and more likely to wear masks (OR 1.02, *p* = 0.02), disinfect (OR 1.03, *p* < 0.01) and take temperature (OR 1.03, *p* < 0.01).

Compared with younger participants, the older participants were less likely depressed (OR 0.97, *p* < 0.01) and anxious (OR 0.98, *p* < 0.01), and more likely to wear masks (OR 1.03, *p* < 0.001), disinfect (OR 1.03, *p* < 0.001) and take temperature (OR 1.01, *p* < 0.001). Compared with females, males were less likely to stay at home (OR 0.62, *p* < 0.001) but more likely to wear masks (OR 1.58, *p* < 0.001). Participants in mild-moderate risk (OR 1.77, *p* = 0.02), moderate risk (OR 2.42, *p* < 0.001), and moderate-high risk (OR 2.11, *p* = 0.02) areas were more likely to wear masks than participants in extremely high-risk areas. Participants with reliable primary sources of information were less likely to be depressed (OR 0.79, *p* = 0.02) and anxious (OR 0.76, *p* < 0.01) than those with unreliable primary sources.

### Mediation analyses via PSS or PTG

Mediation models were conducted with controlling for age, sex, current living area, survey date, and primary source of information. In the models mediated by PSS, the indirect paths were significant for depression, anxiety, and four health-related behaviors (Table 4). As for the PTG, the indirect path was non-significant for home quarantine, though significant for mental health problems and the other three health-related behaviors (Table 4).

**Table 4:** Path coefficients, indirect effects, and 95% bias-corrected confidence interval predicting mental health problems and health-related behaviors (N = 2685)

Path	M = Perceived Social Support Scale				M = Posttraumatic Growth Inventory			
	B	SE	95% CI		B	SE	95% CI	
			Lower	Upper			Lower	Upper
Y = PHQ								
Total effect (c)	− 0.0401*	0.0200	− 0.0794	− 0.0008	− 0.0401*	0.0200	− 0.0794	− 0.0008
Direct effect (c′)	− 0.0227	0.0206	− 0.0630	0.0177	− 0.0128	0.0208	− 0.0537	0.0280
a	0.5073***	0.1332	0.2462	0.7685	0.5693***	0.0824	0.4076	0.7309
b	− 0.0322***	0.0034	− 0.0388	− 0.0256	− 0.0460***	0.0053	− 0.0563	− 0.0356
Indirect effects (ab)	− 0.0163^a^	0.0052	− 0.0270	− 0.0072	− 0.0262^a^	0.0058	− 0.0387	− 0.0161
Y = GAD								
Total effect (c)	0.0032	0.0189	− 0.0339	0.0402	0.0032	0.0189	− 0.0339	0.0402
Direct effect (c′)	0.0191	0.0194	− 0.0190	0.0571	0.0254	0.0195	− 0.0128	0.0636
a	0.5073***	0.1332	0.2462	0.7685	0.5693***	0.0824	0.4076	0.7309
b	− 0.0285***	0.0030	− 0.0344	− 0.0227	− 0.0370***	0.0047	− 0.0462	− 0.0278
Indirect effects (ab)	− 0.0145^a^	0.0046	− 0.0248	− 0.0065	− 0.0211^a^	0.0047	− 0.0305	− 0.0123
Y = Home quarantine								
Total effect (c)	0.0161	0.0219	− 0.027	0.0591	0.0161	0.0219	− 0.0269	0.0591
Direct effect (c′)	0.0112	0.0220	− 0.032	0.0544	0.0129	0.0221	− 0.0304	0.0562
a	0.5073***	0.1332	0.2462	0.7685	0.5693***	0.0824	0.4076	0.7309
b	0.0095**	0.0031	0.0035	0.0156	0.0056	0.0051	− 0.0043	0.0155
Indirect effects (ab)	0.0048^a^	0.0021	0.0014	0.0098	0.0032	0.0031	− 0.0023	0.0096
Y = Mask-wearing								
Total effect (c)	0.1855***	0.0276	0.1314	0.2395	0.1855***	0.0276	0.1314	0.2395
Direct effect (c′)	0.1794***	0.0276	0.1253	0.2335	0.1656***	0.0275	0.1117	0.2196
a	0.5073***	0.1332	0.2462	0.7685	0.5693***	0.0824	0.4076	0.7309
b	0.0147***	0.0034	0.0080	0.0215	0.0292***	0.0055	0.0183	0.0401
Indirect effects (ab)	0.0075^a^	0.0028	0.0028	0.0135	0.0166^a^	0.0042	0.0086	**0.0257**
Y = Disinfection								
Total effect (c)	0.2738***	0.028	0.2188	0.3288	0.2738***	0.028	0.2188	0.3288
Direct effect (c′)	0.2664***	0.0281	0.2113	0.3215	0.2487***	0.0279	0.1940	0.3034
a	0.5073***	0.1332	0.2462	0.7685	0.5693***	0.0824	0.4076	0.7309
b	0.0233***	0.0033	0.0168	0.0298	0.0407***	0.0054	0.0301	0.0513
Indirect effects (ab)	0.0118^a^	0.0037	0.0051	0.0199	0.0232^a^	0.0048	0.0144	0.0334
Y = Temperature-taking								
Total effect (c)	0.3733***	0.0331	0.3084	0.4382	0.3733***	0.0331	0.3084	0.4382
Direct effect (c′)	0.3641***	0.0331	0.2993	0.4289	0.3427***	0.0329	0.2781	0.2072
a	0.5073***	0.1332	0.2462	0.7685	0.5693***	0.0824	0.4076	0.7309
b	0.0221***	0.0036	0.0139	0.0281	0.0404***	0.0059	0.0290	0.0519
Indirect effects (ab)	0.0106^a^	0.0033	0.0048	0.0183	0.0230^a^	0.0047	0.0137	0.0321

Models include controls for age, sex, current living area, survey date, and primary source of information

*p < 0.05; **p < 0.01; ***p < 0.001

^a^Means the indirect effects are significant

### The serial multiple-mediation model

**Fig. 1:**
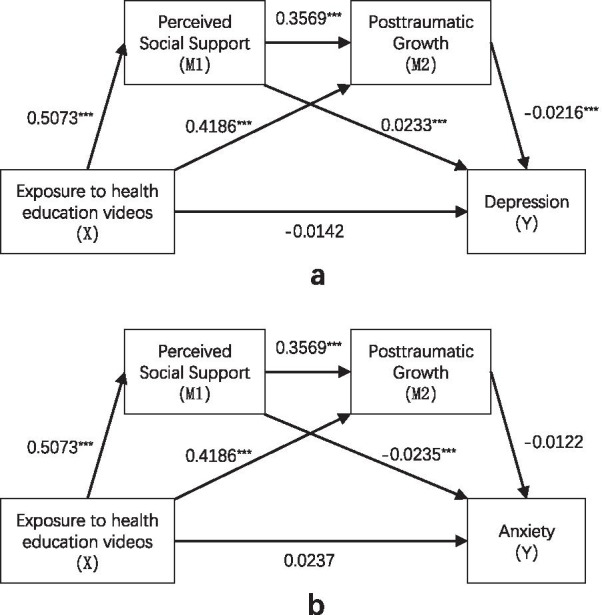
Path diagram illustrating indirect effects and causal paths linking exposure to health education videos with mental health problems. **p < 0.01; ***p < 0.001

**Fig. 2:**
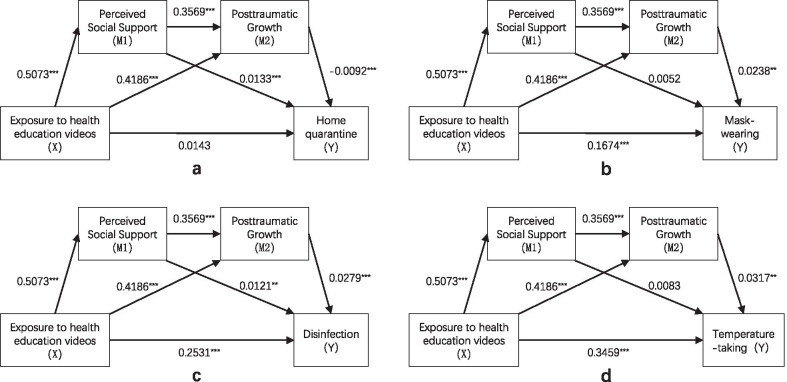
Path diagram illustrating indirect effects and causal paths linking exposure to health education videos with health related behaviors. **p < 0.01; ***p < 0.001

These models assume that exposure to health education videos could increase PTG by increasing PSS, ultimately leading participants to reduce negative emotions and adopt health-related behaviors. Figures 1 and 2 shows serial multiple-mediation models that illustrate indirect effects and causal paths linking exposure to health education videos with mental health problems and health-related behaviors.

For mental health problems, the indirect effects of exposure to health education videos on depression through PSS and PTG were significant (B =  − 0.0046, SE = 0.0021, 95% CI − 0.0098, − 0.0012). The indirect effects of exposure to health education videos on anxiety through PSSS and PTGI were non-significant (B = − 0.0026, SE = 0.0017, 95% CI − 0.0066, 0.0010). For health-related behaviors, the indirect effects of exposure to health education videos on home quarantine through PSSS and PTGI were non-significant (B = − 0.0019, SE = 0.0018, 95% CI − 0.0060, 0.0012). However, the positive indirect effects of exposure to health education videos on the other three health-related behaviors through PSSS and PTGI were significant (Mask-wearing: B = 0.0051, SE = 0.0023, 95% CI 0.0015, 0.0011; Disinfection: B = 0.0059, SE = 0.0024, 95% CI 0.0024, 0.0012; Temperature-taking: B = 0.0067, SE = 0.0026, 95% CI 0.0023, 0.0013).

## Discussion

To our knowledge, this is the first study to evaluate the effect of exposure to health education videos on the public during COVID-19. This study found that the health education videos produced by the School of Public Health of Zhejiang University played a positive role in PSS, PTG, mental health problems and health-related behavior. Specifically, we found that watching health education videos can reduce the risk of anxiety and depression to some extent, and make people more likely to adopt health-promoting behaviors, such as wearing masks, disinfecting and taking temperatures. This finding is consistent with previous studies which indicated exposure to health education videos effectively relieve symptoms of depression and anxiety and contribute to the reduction health-related behaviors that have been modified by mental disorders [12, 28, 29]. One study found that videos from professional and government organizations were the most informative and had the highest quality content [30]. Compared with other institutions, public health schools and academic institutions have a higher understanding of scientific knowledge and distinguish between true and false, so health education videos produced by them are more comprehensive and reliable. In addition, we limit the length of health education videos to about 5 minutes, which is in line with fast-paced lifestyle and the era of short videos in China, enabling audiences to better understand and receive information.

Besides, we found that PSS can decrease depression and anxiety, which is consistent with former studies. A study showed that students who lived alone or had poor relationships with their lovers or friends had higher depression and anxiety scores [31]. Another study targeted at health care workers also found the association between PSS and mental health problems [32]. PSS can promote health-related behaviors as well [33]. We found people with higher PSS were more likely to take temperature, disinfect, and obey home-quarantine. Families and friends may help participants in activities of daily living by providing support on health-related behaviors. As for PTG, we found that it can also decrease depression and anxiety, conforming with former research [34]. Moreover, PTG can lead to more health-related behaviors such as mask-wearing, temperature-taking and disinfection. Using a new positive perspective to analyze the trauma, people could conclude the gains with a more rational attitude. Thus, people were more likely to conduct health-related behaviors.

The most significant finding is exposure to health education videos can affect mental health problems  and behaviors through PTG and PSS. It gives inspiration to possible effective ways of preventing depression and anxiety and promote health-related behaviors during a public health emergency. Firstly, we found that public health videos can significantly improve people’s PSS or PTG, affecting depression, anxiety, and behaviors. Social support is one of the contents in the health education videos. It is conducive to the positive development of social relations via maintaining a high sensitivity to the people around and improving relationships with others [35]. In the video of live streaming chat rooms in Zhejiang University part 2 (how to conduct psychological counseling), we encouraged people to contact relatives and friends or call psychological assistance hotlines. Therefore, people who have watched the video may be more sensitive to social support and establish a more active and intimate relationship with family and friends by increasing self-disclosure and emotional expression. The PTG level reflects the perception of personal benefits of survivors of the traumatic event, including changes in self-perception, relationships with others, and life philosophy, all derived from their efforts to cope with th e trauma consequences [36]. The videos provide evidence-based i nformation and positive perspectives of COVID-19, inspired the public, possibly increasing their PTG. Secondly, according to serial multiple-mediation models, exposure to health education videos can affect mental health problems and behaviors throu gh PTG to PSS. Former studies have found a positive association between social support perception and Postt raumatic Growth Inventory scores. PSS can give people protections against the COVID-19 pandemic. Studies showed that people with higher level PSSS were more likely to change their life positively, leading to PTG [37]. Another study also indicated that support from others could help people shift to a new way and schema when viewing the occurred changes [38]. This study also found that higher PSSS related to more likely PTG, finally leading to the descent of depression. However, this process no longer results in a decrease in anxiety. It may relate to the minor effect of PTG on anxiety compared with PSS. Besides, people with high PSS may also have a lower level of anxiety. It suggested we pay more attention to the considerations and support from our friends and family, helping us develop a more positive perspective and promote health-related behaviors.

### Policy recommendations

Based on the results of this study, we make the following suggestions: Health education videos from public health schools and institutions are effective measures for both psychological intervention and health-related behaviors. PSS and PTG are of vital significance in the psychological resistance and behavioral promotion during pandemics. Therefore, we have the following suggestions to better cope with the challenge of public health emergencies:

1. Under the circumstances of limited daily life, in addition to reporting the epidemic situation and popularizing scientific knowledge, the government also need to pay attention to building good social support networks for the public, such as publishing CDC and psychological consultation hotline in major mainstream media, developing a kind of community service for residents' mutual assistance, and developing the support and service work of social groups etc.
2. Public health schools and institutions should play an active role in producing short videos that are scientific, easy to understand, and lively, and cooperate with the media to expand the scope of publicity and health education.
3. People can improve the level of PTG through mindfulness [39, 40], aerobic exercise [41], and other positive psychological interventions [42–44] to better deal with the negative impact of public health events.

### Limitations

This study has some limitations: first, the demographic data is not comprehensive. Our study did not consider occupation and educational background, and the average age of the entire population was relatively young. Thus, whether this conclusion can be extended to the elderly still needs further investigation. Second, another study indicated that PSS had an indirect effect on PTG [45]. Thus, the association between them still requires further research. Third, as a cross-sectional study, the causality cannot be confirmed. A longitudinal investigation would be valuable.

## Conclusion

Our study found that exposure to health education videos can improve people's perceived social support and the level of post-traumatic growth, help people maintain psychological health, and adopt good health-related behaviors. Therefore, professional colleges or institutions can try to develop a variety of health education videos and increase the interest and influence of these videos to better respond to public health emergencies.

## Supplementary Information

**Additional file 1: Table S1.**Basic information of Zhejiang University science popularization videos and tweet.

## Data Availability

The data used during the current study are available from the corresponding author on reasonable request.

## Abbreviations

COVID-19: Coronavirus Disease 2019
PHQ: Patient Health Questionnaire
GAD: General Anxiety Disorder
PSS: Perceived social support
PTG: Posttraumatic growth
PSSS: Perceived Social Support Scale
PTGI: Posttraumatic Growth Inventory
SPSS: Statistical product and service solutions

## References

[1] Dong L, Bouey J (2020). Public mental health crisis during Covid-19 pandemic, China. Emerg Infect Dis.

[2] Duan L, Zhu G (2020). Psychological interventions for people affected by the Covid-19 epidemic. Lancet Psychiatry.

[3] Zvolensky MJ, Garey L, Rogers AH, Schmidt NB, Vujanovic AA, Storch EA (2020). Psychological, addictive, and health behavior implications of the Covid-19 pandemic. Behav Res Therapy.

[4] Wang C, Pan R, Wan X, Tan Y, Xu L, Ho CS (2020). Immediate psychological responses and associated factors during the initial stage of the 2019 Coronavirus Disease (Covid-19) epidemic among the general population in China. Int J Environ Res Public Health.

[5] Wei Li, Caidi Z, Jinjing L, Zhang Huijuan Wu, Hui YB (2020). Psychological status among different populations during Covid-19 epidemic: a systematic review and meta-analysis. J Tonji Univ (Med Sci).

[6] Feng Z, Liu X, Zhiyi C (2020). Psychological problems among the massive people in Covid-19 pandemic. J Southwest Univ (Soc Sci Ed).

[7] Leung NHL, Chu DKW, Shiu EYC, Chan KH, McDevitt JJ, Hau BJP (2020). Respiratory virus shedding in exhaled breath and efficacy of face masks. Nat Med.

[8] Chen P, Mao L, Nassis GP, Harmer P, Ainsworth BE, Li F (2020). Coronavirus Disease (Covid-19): the need to maintain regular physical activity while taking precautions. J Sport Health Sci.

[9] Hao L (2012). The important role of medical science popularization in responding to public health emergencies. Strait J Prev Med.

[10] Bieri FA, Gray DJ, Raso G, Li YS, McManus DP (2012). A systematic review of preventive health educational videos targeting infectious diseases in schoolchildren. Am J Trop Med Hyg.

[11] Dahodwala M, Geransar R, Babion J, de Grood J, Sargious P (2018). The impact of the use of video-based educational interventions on patient outcomes in hospital settings: a scoping review. Patient Educ Couns.

[12] Tuong W, Larsen ER, Armstrong AW (2014). Videos to influence: a systematic review of effectiveness of video-based education in modifying health behaviors. J Behav Med.

[13] Hall BJ, Xiong YX, Yip PSY, Lao CK, Shi W, Sou EKL (2019). The association between disaster exposure and media use on post-traumatic stress disorder following Typhoon Hato in Macao, China. Eur J Psychotraumatol.

[14] Veronese G, Pepe A (2019). Measurement and evaluation in counseling and development using the posttraumatic growth inventory-short form with Palestinian helpers living in conflict areas. Meas Eval Couns Dev.

[15] Jang S-H, Lee H-R, Yeu H-N, Choi S-O (2014). The effects of posttraumatic growth and meaning in life on health promotion behavior in cancer patients. Asian Oncol Nurs.

[16] Tedeschi RG, Cann A, Taku K, Senol-Durak E, Calhoun LG (2017). The posttraumatic growth inventory: a revision integrating existential and spiritual change. J Trauma Stress.

[17] Tamiolaki A, Kalaitzaki AE (2020). "That which does not kill us, makes us stronger": Covid-19 and posttraumatic growth. Psychiatry Res.

[18] Xu J, Ou J, Luo S, Wang Z, Chang E, Novak C (2020). Perceived social support protects lonely people against Covid-19 anxiety: a three-wave longitudinal study in China. Front Psychol.

[19] Xing H, Yu W, Xu F, Chen S (2017). Influence of social support and rearing behavior on psychosocial health in left-behind children. Health Qual Life Outcomes.

[20] Choe MA, Yi M, Choi JA, Shin G (2012). Health knowledge, health promoting behavior and factors influencing health promoting behavior of North Korean defectors in South Korea. J Korean Acad Nurs.

[21] Turner-Musa J, Lipscomb L (2007). Spirituality and social support on health behaviors of African American undergraduates. Am J Health Behav.

[22] Fang L, Chuang DM, Al-Raes M (2019). Social support, mental health needs, and HIV risk behaviors: a gender-specific, correlation study. BMC Public Health.

[23] Wu C, Liu Y, Ma S, Jing G, Zhou W, Qu L (2021). The mediating roles of coping styles and resilience in the relationship between perceived social support and posttraumatic growth among primary caregivers of schizophrenic patients: a cross-sectional study. BMC Psychiatry.

[24] Cheng K, Qiuyan C (2011). A study on the relationship between posttraumatic growth and perceived social support among middle school students in earthquake-stricken areas. J Southwest Univ Natl (Humanit Soc Sci).

[25] Kroenke K, Spitzer RL, Williams JB (2001). The Phq-9: validity of a brief depression severity measure. J Gen Intern Med.

[26] Spitzer RL, Kroenke K, Williams JB, Löwe B (2006). A brief measure for assessing generalized anxiety disorder: the Gad-7. Arch Intern Med.

[27] Zimet GD, Powell SS, Farley GK, Werkman S, Berkoff KA (1990). Psychometric characteristics of the multidimensional scale of perceived social support. J Pers Assess.

[28] Liu Y, Chen J, Pan Y, Cai Y, Ge C, Chu H, et al. The effects of video based nursing education on perioperative anxiety and depression in patients with gastric cancer. Psychol Health Med. 2021;26(7):867–76. 10.1080/13548506.2020.1825756 33044837

[29] Hua L, Yingjuan L, Jingshu Z, Wei C (2014). The effect of health education video on ocular massage after trabeculectomy. Comput Inform Nurs: CIN.

[30] Li HO, Bailey A, Huynh D, Chan J (2020). Youtube as a source of information on Covid-19: a pandemic of misinformation?. BMJ Glob Health.

[31] Shao R, He P, Ling B, Tan L, Xu L, Hou Y (2020). Prevalence of depression and anxiety and correlations between depression, anxiety, family functioning, social support and coping styles among chinese medical students. BMC Psychol.

[32] Hou T, Zhang T, Cai W, Song X, Chen A, Deng G (2020). Social support and mental health among health care workers during Coronavirus Disease 2019 outbreak: a moderated mediation model. PLoS ONE.

[33] Faleschini S, Millar L, Rifas-Shiman SL, Skouteris H, Hivert MF, Oken E (2019). Women's perceived social support: associations with postpartum weight retention, health behaviors and depressive symptoms. BMC Womens Health.

[34] Walsh DMJ, Groarke AM, Morrison TG, Durkan G, Rogers E, Sullivan FJ (2018). Measuring a new facet of post traumatic growth: development of a scale of physical post traumatic growth in men with prostate cancer. PLoS ONE.

[35] Dakof GA, Taylor SE (1990). Victims' perceptions of social support: what is helpful from whom?. J Pers Soc Psychol.

[36] Tedeschi RG, Calhoun LG (1996). The posttraumatic growth inventory: measuring the positive legacy of trauma. J Trauma Stress.

[37] Ekim A, Ocakçi A (2015). Relationship between posttraumatic growth and perceived social support for adolescents with cancer. J Hosp Palliat Nurs.

[38] Tedeschi R, Calhoun L (2004). Posttraumatic growth: conceptual foundations and empirical evidence. Psychol Inq.

[39] Accoto A, Chiarella SG, Raffone A, Montano A, de Marco A, Mainiero F (2021). Beneficial effects of mindfulness-based stress reduction training on the well-being of a female sample during the first total lockdown due to Covid-19 pandemic in Italy. Int J Environ Res Public Health.

[40] Garland EL, Farb NA, Goldin P, Fredrickson BL (2015). Mindfulness broadens awareness and builds eudaimonic meaning: a process model of mindful positive emotion regulation. Psychol Inq.

[41] Ping Y (2017). Influence of aerobic exercise combined with continuity of care on post-traumatic growth and psychological resilience of breast cancer patients. Nurs Res China.

[42] Cui PP, Wang PP, Wang K, Ping Z, Wang P, Chen C (2021). Post-Traumatic growth and influencing factors among frontline nurses fighting against Covid-19. Occup Environ Med.

[43] Ey S, Soller M, Moffit M (2020). Protecting the well-being of medical residents and faculty physicians during the Covid-19 pandemic: making the case for accessible, comprehensive wellness resources. Glob Adv Health Med.

[44] Li L, Mao M, Wang S, Yin R, Yan H, Jin Y, et al. Posttraumatic growth in Chinese nurses and general public during the Covid-19 outbreak. Psychol Health Med. 2021:1–11. 10.1080/13548506.2021.1897148 33726576

[45] Li Y, Bai H, Lou F, Cao F (2019). A conceptual model of posttraumatic growth of nursing students with a disabled parent. Int J Nurs Sci.

