# Supplementary material for: The impact of health education videos on general public’s mental health and behavior during COVID-19
Source: Glob Health Res Policy. 2021 Sep 30;6:37. doi: 10.1186/s41256-021-00211-5 (PMC8482355; doi:10.1186/s41256-021-00211-5)
Supplement: Supplementary file 1 — Additional file 1: Table S1.Basic information of Zhejiang University science popularization videos and tweet. [file 41256_2021_211_MOESM1_ESM.docx]

**Supplementary Table S1 Basic information of Zhejiang University science popularization videos and tweet**

| videos/tweet | Title of the videos/tweet | main content | duration | websites |
| --- | --- | --- | --- | --- |
| video1 | Tips on prevention and control of COVID-19 | The transmission route and characteristic of COVID-19 (spread fast, toxicity gets weaker), prevention advice (frequently ventilate, avoid close distance, sneezing, etc.), the distinction with SARS (COVID-19 toxicity is lower), present hormone treatment uses. | 1m22s | https://wap.peopleapp.com/article/rmh11075319/rmh11075319?from=timeline&isappinstalled=0 |
| video2 | These small details help you stay away from COVID-19 | Introduce home isolation, wearing masks and its correct way, disinfection, frequently wash hands and six aspects of the spread of rumors. | 1m8s | https://www.bilibili.com/video/av86155888/?redirectFrom=h5 |
| video3 | It is finally here! It's my turn to stay at home to prevent the spread of COVID-19! | Introduce the online consultation service and the detailed medical procedures for people with fever. People are advised to quarantine at home, reduce going out, and cooperate with community prevention and control. | 2m36s | http://zj.people.com.cn/n2/2020/0203/c187103-33759421.html |
| video4 | Live streaming chat rooms in Zhejiang University part.1 (how to wear a mask and wash hands) | Introduce the types of masks available in the market, how to properly wear and dispose of masks, daily ventilation and disinfection, seven-step of washing hands. | 5m7s | https://mp.weixin.qq.com/s/_Gh7S8OjuCSrCYyrokdR_g |
| video5 | Live streaming chat rooms in Zhejiang University part.2 (how to conduct psychological counseling) | Introduce simple and practical methods to relieve anxiety during the epidemic: slow movement (eating and speak slowly, deep and slow breathing); distinguish real information from fake one; Regular work and rest; Healthy diet; Indoor exercise (butterfly paddle); Divert attention appropriately; Psychological Assistance Hotline | 3m11s | https://t.cn/A6Prkm4P |

| videos/tweet | Title of the videos/tweet | main content | duration | websites |
| --- | --- | --- | --- | --- |
| video6 | Show responsibility of the people in the face of COVID-19（Dialect version of epidemic prevention video） | Invite the elderly to give guidance on COVID-19 for the public with dialect: stay at home and stop party; wear a mask and wash hands frequently; report whereabouts initiatively; stay in a peaceful mood. | 59s | https://wap.peopleapp.com/article/rmh11239025/rmh11239025?from=singlemessage&isappinstalled=0 |
| video7 | How to eat healthily during COVID-19? | Form healthy diet habits (wash hands, pay attention to the dining hygiene), ensure food safety (fasting wild animals, eat cooked food and boiled water), strengthen nutrition supplement and improve immunity (balanced food, more fresh fruits and vegetables, guaranteed protein intake, appropriate nutrition agent) | 5m8s | https://zj.zjol.com.cn/news.html?id=1384455 |
| video8 | Work together to prevent and control the epidemic | A TV interview with Xifeng Wu, the dean of the School of Public Health, Zhejiang University in this program. The contents include the difference between COVID-19 and SARS, prevention and control difficulty, advice to the citizens, the role of big data technology in the epidemic, the interpretation of public health policy and the present situation of epidemic prevention and control. | 34m40s | https://weibo.com/1727386613/IsjIkwY0v |
| tweet | 50 key issues for prevention and control of COVID-19 | COVID-19 and its pathogens, the source of infection and transmission, population susceptibility and protection (home isolation, wearing masks, disinfection, treatment of fever and cough symptoms, etc.), the CDC consultation telephone number in Zhejiang and epidemic rumors query websites. | / | https://mp.weixin.qq.com/s/2zRdll-ojpwnylIQSlHXQg |
